# Multiplex lithography for multilevel multiscale architectures and its application to polymer electrolyte membrane fuel cell

**DOI:** 10.1038/ncomms9484

**Published:** 2015-09-28

**Authors:** Hyesung Cho, Sang Moon Kim, Yun Sik Kang, Junsoo Kim, Segeun Jang, Minhyoung Kim, Hyunchul Park, Jung Won Bang, Soonmin Seo, Kahp-Yang Suh, Yung-Eun Sung, Mansoo Choi

**Affiliations:** 1 Global Frontier Center for Multiscale Energy Systems, Seoul National University, Seoul 151-744, Korea; 2 Department of Mechanical and Aerospace Engineering, Seoul National University, Seoul 151-744, Korea; 3 Division of WCU Multiscale Mechanical Design, Department of Mechanical and Aerospace Engineering, Seoul National University, Seoul, 151-742, Korea; 4 Center for Nanoparticle Research, Institute for Basic Science (IBS), Seoul 151-742, Korea; 5 School of Chemical and Biological Engineering, Seoul National University, Seoul 151-742, Korea; 6 Energy Harvesting Devices Research Section, Electronics and Telecommunications Research Institute, Daejeon 305-700, Korea; 7 Center for Materials Architecturing, Korea Institute of Science and Technology, Seoul 136-791, Korea; 8 College of BioNano Technology, Gachon University, Gyeonggi 461-701, Korea

## Abstract

The production of multiscale architectures is of significant interest in materials science, and the integration of those structures could provide a breakthrough for various applications. Here we report a simple yet versatile strategy that allows for the LEGO-like integrations of microscale membranes by quantitatively controlling the oxygen inhibition effects of ultraviolet-curable materials, leading to multilevel multiscale architectures. The spatial control of oxygen concentration induces different curing contrasts in a resin allowing the selective imprinting and bonding at different sides of a membrane, which enables LEGO-like integration together with the multiscale pattern formation. Utilizing the method, the multilevel multiscale Nafion membranes are prepared and applied to polymer electrolyte membrane fuel cell. Our multiscale membrane fuel cell demonstrates significant enhancement of performance while ensuring mechanical robustness. The performance enhancement is caused by the combined effect of the decrease of membrane resistance and the increase of the electrochemical active surface area.

## Abstract (web summary)

Multiplex lithography is a technique that can be used to fabricate complex soft materials. Here, the authors develop a method to prepare multilevel multiscale structures and demonstrate its application on polymer electrolyte membrane fuel cells which display decreased membrane resistance and increased electrochemical active surface area.

## Graphical abstract

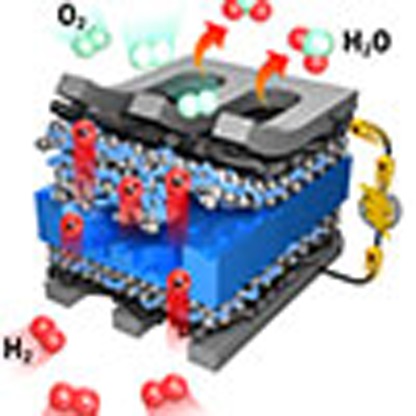

Soft lithography, a rapid prototyping of structures geared for both the microscale and nanoscale, is a collection of versatile techniques that allow for the development of novel structures and their incorporation into advanced applications with the aid of flexible, elastomeric stamps^1^. Such a flexibility, which is an inherent property of soft materials, has significantly improved technologies in the fields of artificial sensors^2^,^3^,^4^,^5^, wearable electronics^6^,^7^, and energy and optical devices^8^,^9^,^10^. In addition, it has accelerated new capabilities for real-actuating and soft robotics^11^,^12^. This appealing strategy, which is based on the softness of materials, generally imposes a certain degree of elastic/plastic deformation when external forces are applied, thereby providing the potential for achieving complex, hierarchical engineering^13^. For example, rational mechanical deformations, including the stretching^4^, bending^13^ and bulging^3^ of elastomers and chemical modifications for surficial instabilities on given surfaces^14^,^15^,^16^, have been widely demonstrated to obtain complex structures.

Complex hierarchical microarchitectures have emerged^17^ to overcome engineering issues arisen from a single scale^18^,^19^,^20^. However, the production of complex multilevel and multiscale architectures with soft materials remains challenging, mainly because soft lithography basically employs sufficient thermal or ultraviolet treatments to fully solidify the raw materials for high pattern fidelity^14^,^21^,^22^,^23^,^24^. Ultraviolet-curable resins that guarantee simple yet rapid replication within minutes at both the microscale and nanoscale are fully solidified under a crosslinking with polymer chains by ultraviolet irradiation. Therefore, further processing such as imprinting or bonding after the solidification is difficult and the integration of the structures to achieve multilevel multiscale architectures is a challenging task. To address these challenges, we developed a multiplex lithography method that utilizes oxygen inhibition effects on ultraviolet-curable resin by controlling the spatial distribution of oxygen concentration in the resin. The deliberate spatial control of oxygen concentration in the resin allows us to enable selective imprinting and bonding on each side of a membrane, which leads to LEGO-like multiplex stacking of micrometre membranes together with the formation of multiscale patterns. Via the method, multilevel multiscale Nafion membranes were prepared and applied to polymer electrolyte membrane fuel cells (PEMFCs). Our multiscale membrane-based PEMFC not only demonstrates significant enhancement of the fuel cell performance but also ensures mechanical robustness. The performance enhancement is attributed to the combined effect of the decrease of membrane resistance and the increase of the electrochemical active surface area (ECSA).

## Results

### Multiplex lithography utilizing oxygen inhibition effect

**Figure 1:**
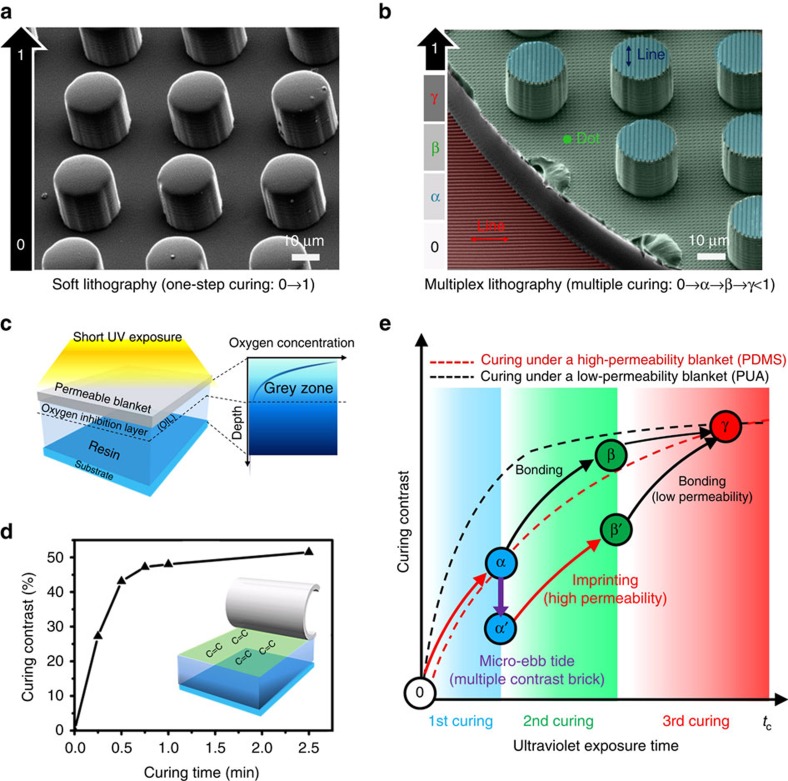
Concept of multiplex lithography utilizing oxygen inhibition effect. (**a**) Single-scale structure from typical soft lithography. (**b**) Multilevel multiscale architecture made from multiplex lithography. The architecture has independent nanopatterns (dots and lines) on each flat surface. (**c**) Illustration of a ‘grey zone' with infiltrated oxygen from a permeable PDMS blanket. (**d**) Curing contrast variations after time-dependent ultraviolet exposure. (**e**) Variations of curing contrast with ultraviolet exposure time to form complex hierarchical architectures via multiplex lithography.

The basic concept of multiplex lithography is illustrated in Fig. 1. Typical soft lithography for single-scale structures usually uses a one-step curing process with a mother mould (Fig. 1a), which could not manufacture complex multilevel multiscale architectures such as the one shown in Fig. 1b, that was fabricated from our multiplex lithography. Figure 1b shows our multilevel architecture containing vertical, parallel nanolines and nanodots placed on different levels (blue lines on the third level, red ones on the first level and green dots on the second level). This unique structural hierarchy is derived by utilizing the scavenging effect of oxygen infiltrated through a highly permeable polydimethyl siloxane (PDMS) blanket, which results in a thin layer or ‘grey zone' that contains the infiltrated oxygen and inhibits radical-induced polymerizations^25^ (Fig. 1c; Supplementary Fig. 1). This region is called oxygen inhibition layer (OIL). Under the ultraviolet irradiation, a gradual curing of the polyurethane acrylate (PUA) resin occurs in OIL near the top surface (Fig. 1d; Supplementary Fig. 2).^26^ According to the experiments we performed, this process remains partially cured viscoelastic surface layer on top of PUA brick with a 30–55% curing contrast (CC; defined in the Method section) depending on curing time. As shown in the inset of Fig. 1d, this viscoelastic surface layer of PUA contains many crosslinkable bonds enabling the imprinting of specific patterns if it has a low CC such as 30–45% or the bonding if it has an intermediate CC such as 50–60%. For a fixed irradiation time, the CC could be controlled by changing oxygen concentration in the resin. For example, the part of a resin containing higher oxygen concentration should have a lower CC due to more severe oxygen inhibition effect. If the top part of the resin is designed to have a high oxygen concentration while the bottom part has an intermediate level of concentration, then the imprinting is possible on the top part while the bonding is possible on the bottom part. Such a brick having different CC at each side of the resin brick is called as a multiple contrast brick (MCB) in this study and the synthesis of MCB is the first step of our multiplex lithography. The key to the fabrication of MCB is how the oxygen concentration could be controlled in different spatial regions of the same resin brick, which will be explained later. The construction of MCB together with multiple curing steps enables the stacking of micrometre membranes having multiscale patterns, which is analogous to LEGO integrating procedure leading to multilevel multiscale structures. We found that such a stacking procedure for bonding and imprinting needs high pressure (∼20 N cm^−2^) onto the membrane.

Figure 1e describes the variations of CC during the process of multiplex lithography. Dashed red curve shows a slow increase of CC with exposure time when the curing occurs under a high-oxygen-permeable blanket such as a PDMS, while the dashed black curve shows a fast curing under a low-permeability blanket such as a PUA. This is easily understood from the oxygen penetration through the blanket and its inhibition effect. During the first curing step under top and bottom PDMS blankets, CCs both at the top and bottom parts of the PUA resin reach the same level-*α*. Then, in our method, only the top part of the resin could be forced to contain higher oxygen concentration so that CC becomes *α*′ using micro-ebb tide (will be explained later), while the bottom part remains the same as *α*. This resin brick having different CCs at the top and bottom is the MCB as defined earlier. Then, the imprinting is performed at the top part of MCB while the bonding (or stacking) of the other membrane is performed at the bottom, which finally yields multilevel multiscale structures.

### Procedures to fabricate multilevel multiscale architectures

**Figure 2:**
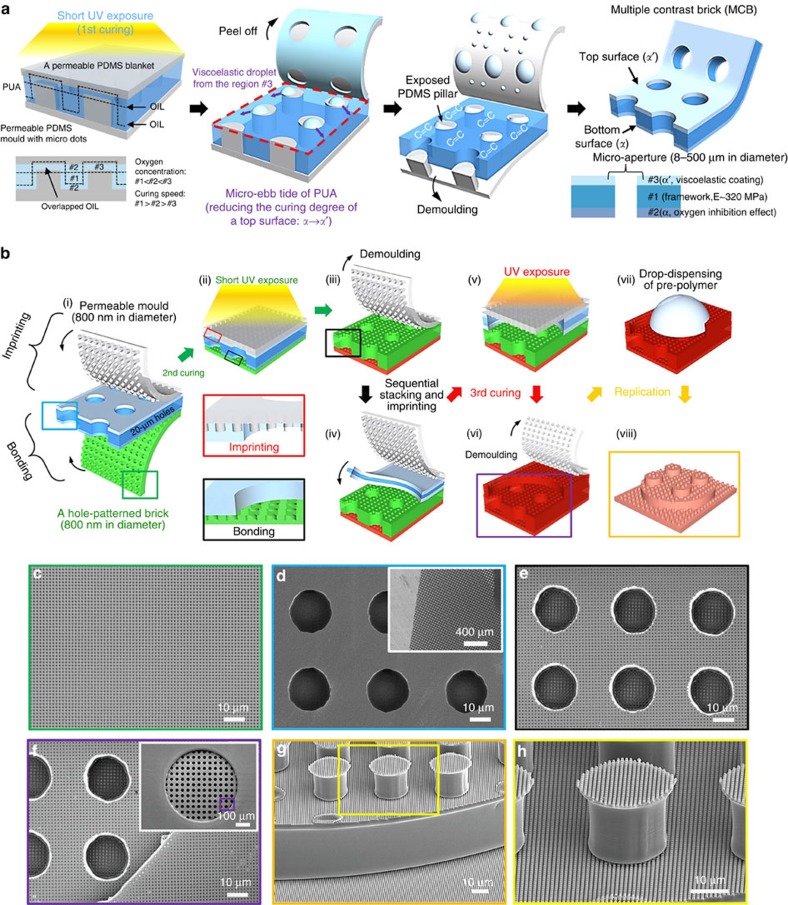
Multiple contrast bricks and multilevel multiscale architectures. (**a**) Schematic illustration for the overlapping of OIL and the resulting micro-ebb tide after the first ultraviolet exposure. The micro-ebb tides gradually reduce the curing contrast on the top surface of the brick from *α* to *α*′. (**b**) Schematic illustration of the multiplex lithography process by vertical stacking and imprinting each multiple contrast bricks (MCB). Both imprinting and bonding are achieved with the top and bottom surfaces of the brick (*α*′ and *α*, respectively) to form complex hierarchical architectures. (**c**–**h**) SEM images of the bricks (**c**,**d**), monolithic assemblies (**e**,**f**) and final architectures after replication (**g**,**h**). A brick with 20-μm holes (**d**) was interconnected with a bottom mould with 800-nm holes (**e**), whereas the top surface of the brick was imprinted with nanopatterns during the integration. After a sequential interconnection with a brick with 500-μm holes was performed (**f**), a three-level multiscale structure with complex hierarchy was obtained from the polymer (**g**,**h**) via replication.

The first step of multiplex lithography is the fabrication of a MCB by slightly curing the PUA resin between two permeable PDMS layers (bottom: PDMS mould with microdots, top: flat PDMS blanket in the left panel of Fig. 2a). At the top part of the resin, an overlapped OIL having high oxygen concentration within thin PUA resin is formed due to oxygen penetrations both from top and bottom PDMS layers. Within the sandwich-like moulding set-up, three distinct regions including overlapped OIL (#3) are produced to have different oxygen concentrations in PUA resin (oxygen concentrations #1<#2<#3, in other term, curing speed #1>#2>#3). The simulation of oxygen diffusion through this set-up was conducted and correctly predicted OIL and three different regions (Supplementary Fig. 3; Supplementary Methods)

After an initial short ultraviolet treatment as a first curing, the uncured PUA resin in the overlapped OIL experiences a spontaneous dewetting on top of PDMS pillar during the process of PDMS peeling off and becomes viscoelastic droplets due to the low affinity on the exposed hydrophobic PDMS surface. Then these uncured PUA droplets escape from PDMS surface and move to neighbouring PUA surface leading to coat the PUA surface (red dashed area of Fig. 2a), which we call the process as micro-ebb tide of PUA (see more details in Supplementary Notes on observation of the micro-ebb tides after the first ultraviolet curing, Supplementary Fig. 4 and Supplementary Movies 1 and 2). This coating process with uncured PUA resin droplets results in a marked decrease in the curing contrast (CC from *α* to *α*′) at the top surface because droplets placed initially at overlapped OIL regions (#3) should have high oxygen concentration that strongly inhibits the ultraviolet reaction while the bottom part maintains the same CC of *α* (see the right panel of Fig. 2a). The decrease in CC from *α* to *α*′ after first curing, which was shown in Fig. 1e, was describing this process. The demoulding process of the bottom PDMS mould completes the fabrication of MCB having the lowest CC at the top, the intermediate CC at the bottom and the highest CC in the middle (see the rightmost panel of Fig. 2a). This MCB is the unit cell of basic building block in our LEGO-like multiplex process. Various MCBs were demonstrated to be fabricated in Supplementary Figs 5 and 6.

Figure 2b depicts the LEGO-like multiplex process. The MCB has two different curing contrasts on both sides (CC ∼*α* and *α*′). Monolithic integration (or bonding) is performed on the bottom of the brick having CC ∼*α* (CC ∼55%) by placing it onto a pre-patterned PUA having 800-nm holes with CC ∼*β* (CC ∼60%) while imprinting is done on the top having *CC* ∼*α*′ (CC ∼45%) by pressing permeable PDMS mould having 800-nm pillars. The SEM image in Fig. 2c shows 800-nm-hole patterned (depth: 800 nm) PUA surface and Fig. 2d shows the top view of MCB having 20-μm holes. During the second ultraviolet exposure (Fig. 2bii), the top part of MCB with an initial low CC (*α*′) allows imprinting and is further cured to result in the intermediate CC (*β′*) with a slow curing speed shown as a red curve in Fig. 1e under the high-permeable PDMS mould. The demoulding of PDMS mould completes the imprinting (Fig. 2biii). Note that the imprinted part of PUA still has an intermediate CC ∼*β′* even after the second ultraviolet exposure. On the other hand, at the bottom part of MCB with an intermediate CC∼*α* experiences bonding and during ultraviolet exposure, the bottom part also becomes cured further, but follows the black curve (higher curing speed) shown in Fig. 1e under the low-permeable PUA mould. The CC of the bottom part eventually reaches *β* after complete bonding between MCB and PUA brick occurs. The black box of Fig. 2biii shows the completed two-level imprinted PUA membrane and Fig. 2e illustrates its top SEM image that clearly shows imprinted 800-nm holes on the upper level of 20-μm hole containing PUA brick and at the same time, 800-nm holes on the lower level inside 20-μm hole. Since the imprinted part of two-level membrane still has an intermediate level of CC (*β′*), bonding of the other MCB is possible. In Fig. 2biv, another MCB with 500-μm hole is placed on already made two-level imprinted structure and sandwiched by the PDMS mould having 800-nm pillars on the top for imprinting. Third ultraviolet exposure (Fig. 2bv) and demoulding of the PDMS mould complete three-level multiscale structure having 500-, 20-μm and 800-nm scale patterns shown as LEGO-like red brick (Fig. 2bvi). The SEM image in Fig. 2f not only confirms 800-nm imprinted holes on third and second levels but also shows bonded original PUA brick having 800-nm holes inside 20-μm holes. As a final step, we performed a replica moulding with various polymers (Fig. 2bvii) to form a three-level multiscale architecture (Fig. 2bviii). Its SEM images are shown in Fig. 2g,h. Various multilevel multiscale structures were fabricated via the method (see more details in Supplementary Notes on vertical stacking of MCBs and observation of the interconnections and Supplementary Figs 7–9) including four-level multiscale architecture (see Supplementary Notes on rapid prototyping of multilevel hierarchical structures via serial stacking procedures and Supplementary Fig. 10)

### Fabrication of three-level multiscale Nafion membranes

**Figure 3:**
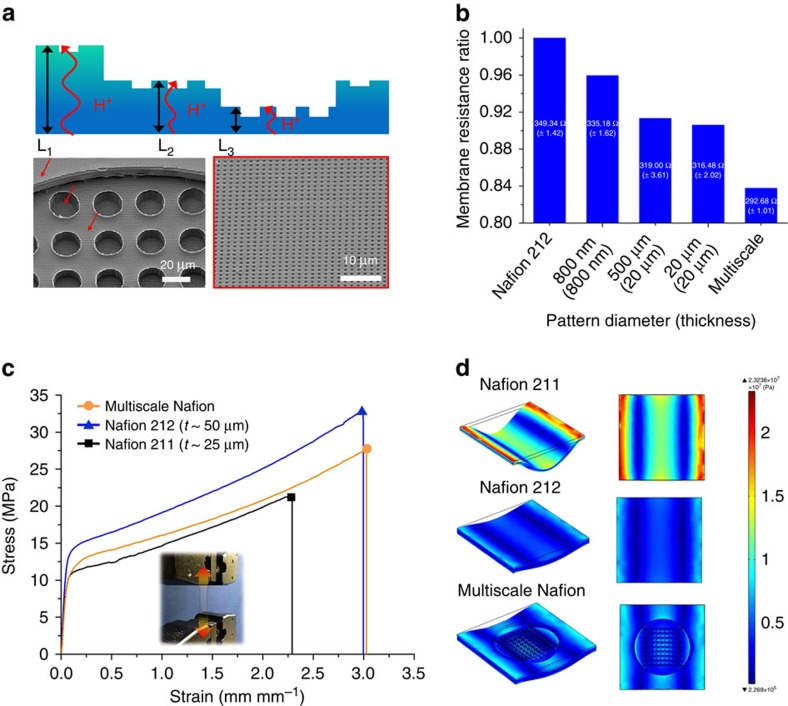
Multiscale Nafion membrane and its properties. (**a**) Schematic illustration of the multiscale Nafion membrane and SEM images of imprinted multiscale Nafion membrane. (**b**) Measured membrane resistance of Nafion membranes having different patterns more than five times. (**c**) Stress–strain tests of Nafion membranes more than five times. (**d**) Simulation for stress distribution onto the membranes.

We constructed a multiscale PEMFC by incorporating multilevel multiscale architectures into a Nafion membrane that selectively transports protons (H^+^) from the anode to the cathode in the PEMFC (Fig. 3a). The development of a high-performance electrode materials^27^,^28^,^29^,^30^,^31^, membrane^32^,^33^ and novel structure for a membrane electrode assembly (MEA)^34^,^35^ have been extensively studied over several decades. Of these advancements, approaches for reducing the resistance of a Nafion membrane have been explored, including lowering the thickness of the membrane^36^. However, such a reduction of Nafion thickness remains challenging and impractical due to the low mechanical properties of the thin Nafion membrane^37^. Here we demonstrate novel multilevel and multiscale Nafion membranes to satisfy both requirements of low membrane resistance and sufficient mechanical robustness (Fig. 3a; Supplementary Fig. 11). Figure 3b shows the reduced membrane resistance in accordance with the different patterns on the Nafion membrane. Structurally, the multilevel Nafion membrane consists of three different thicknesses (*L*_1_>*L*_2_>*L*_3_), where *L*_1_ is the original thickness of the membrane (∼50 μm; Fig. 3a). As a thin Nafion membrane is often fragile during the catalyst-coating process (Supplementary Fig. 12), *L*_1_ provides a geometrical reinforcement from the hierarchical levelling of relatively thin areas with a thickness of *L*_2_ (∼30 μm) and *L*_3_ (∼10 μm)^18^. In this way, both regions (*L*_2_ and *L*_3_) affected the reduced membrane resistance, while the region of *L*_1_ ensured mechanical robustness. From the three-level multiscale architecture, we reduced the membrane resistances of Nafion by ∼16% from the original Nafion membrane (Fig. 3b).

We carried out stress–strain measurements to examine the mechanical strength of our multiscale Nafion membrane and compared with the conventional flat cases, Nafion 212 membrane with thickness of ∼50 μm, and Nafion 211 membrane with thickness of ∼25 μm (Fig. 3c). The results show that the multiscale Nafion membrane has only ∼12% lower tensile strength than the Nafion 212 membrane (∼31.11 MPa) but ∼16 % higher maximum tensile strength than the Nafion 211 membrane (∼23.55 MPa). Elongation before break for our multiscale case is in the same range as that of 50 μm Nafion 212. Measured tensile strengths of Nafion 211 and 212 agree well with the values provided by the manufacturer (DuPont). These measurements support our idea of using multilevel multiscale membrane, which not only provides a lower proton resistance but also ensures the mechanical robustness. A thinned but flat membrane like Nafion 211 could lower the membrane resistance but could not provide sufficient mechanical strength as evidenced in Fig. 3c and Supplementary Table 1. In addition, we did a simulation study when applying the same force perpendicular to the membrane surface to examine whether the membrane could withstand during the process of spraying Pt/C. The simulation results in Fig. 3d show that the multiscale Nafion membrane (thickness of ∼10 μm in certain areas) yields almost the same level of stress distribution as that of Nafion212 (*t* ∼50 μm), while the case of Nafion211 (t ∼25 μm) shows high stress concentration region near side grips. This result supports the robustness of our multiscale membrane.

### Multiscale polymer electrolyte membrane fuel cells

**Figure 4:**
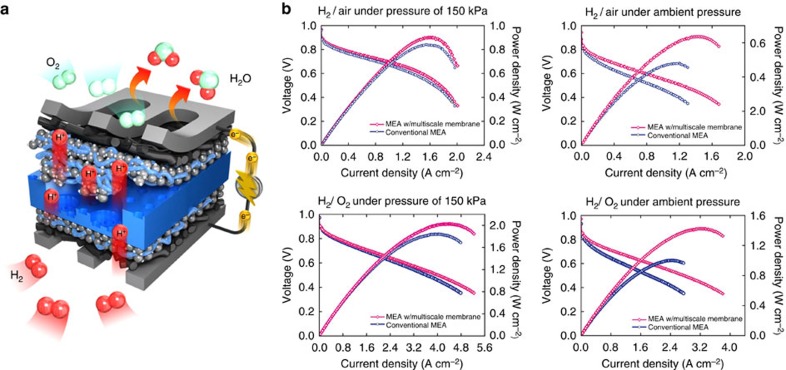
Performance of multiscale polymer electrolyte membrane fuel cells. (**a**) Schematic illustration of the device operation with multiscale Nafion membrane. (**b**) Polarization curves of conventional membrane electrode assembly (MEA) and the MEA with a multiscale Nafion membrane under the conditions of H_2_/Air and H_2_/O_2_ with or without outlet pressure.

We constructed PEMFCs by spraying Pt/C catalysts onto both sides of the Nafion membrane (Pt loading: 0.12 mg cm^−2^, see Supplementary Fig. 13). When operating the MEA with our multiscale membrane in a fully humidified condition of H_2_/O_2_ (or H_2_/air), this MEA exhibited much improved performance compared with a conventional one as shown in Fig. 4b (Supplementary Fig. 14; Supplementary Table 2). The MEA with a multiscale membrane yielded the maximum power density of up to 2.026 W cm^−2^ under additional pressure (150 kPa) following the conditions of the US Department of Energy (DOE)^34^,^38^. The maximum power density increment of ∼42.3% was achieved in the case of H_2_/O_2_ conditions under ambient pressure. The enhancement is mainly due to the aforementioned reduced membrane resistance and the enlarged electrochemically active area, which is dependent but not proportionally increased by the increment of geometrical surface area of Nafion membranes (Supplementary Fig. 15). For the pressurized case, we found that there was a smaller performance enhancement by about 10% compared with the flat membrane case.

## Discussion

**Figure 5:**
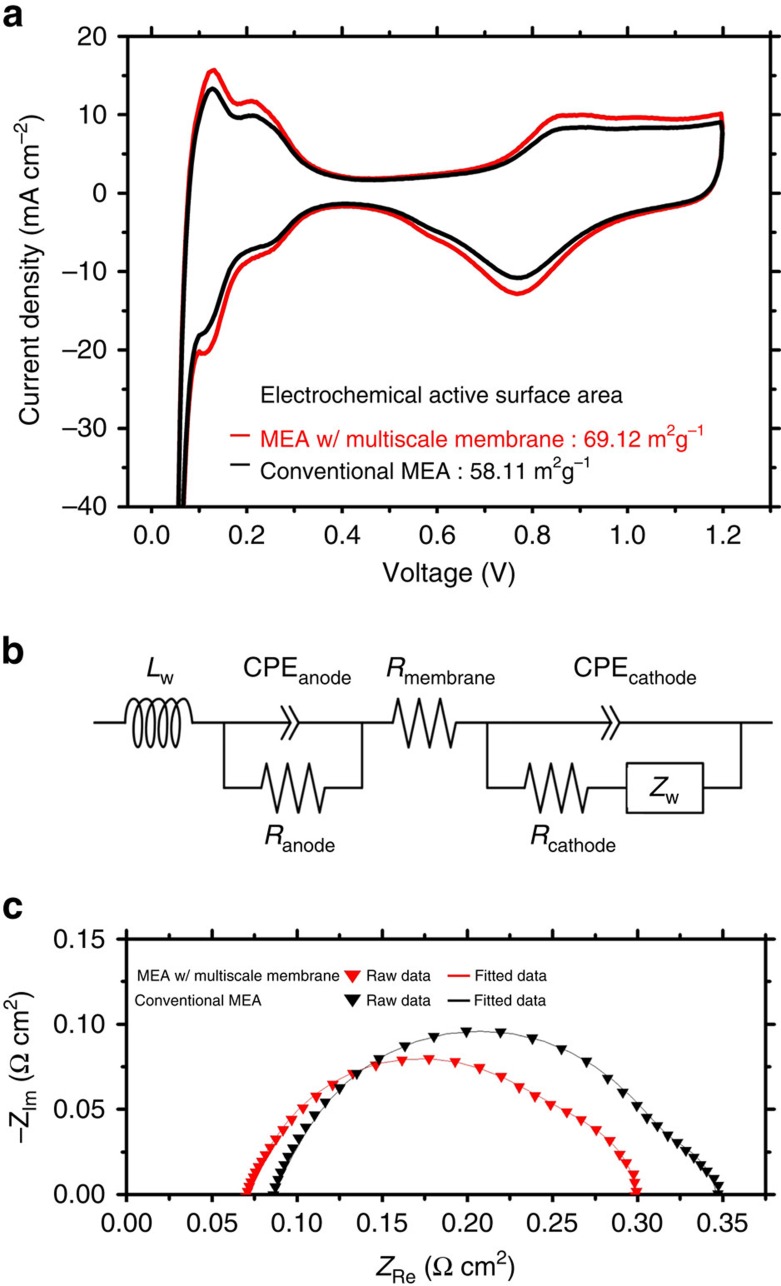
Electrochemical analysis for a single cell. (**a**) Cyclic voltammogram (CV) of the cathode catalyst layers of a conventional MEA and an MEA with a multiscale membrane. The electrochemical active surface area (ECSA) was calculated as follows: 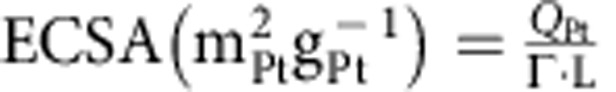, where *Q*_Pt_ is the charge density of Pt measured from the CV in the range of the proton desorption region (mC m^−2^), Г is the charge required to reduce a monolayer of protons adsorbed on the Pt surface, 2100 
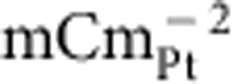
, and *L* is the Pt loading in the cathodes, g_Pt_ m^−2^. (**b**) Equivalent circuit of the PEMFC (*L*_W_=inductance of the electric wire, *R*_membrane_=internal membrane resistance, *R*_cathode (anode)_=charge transfer resistance of the cathode (anode), CPE_cathode (anode)_=constant phase element of the cathode (anode) and *Z*_W_=Warburg impedance). (**c**) Electrochemical impedance spectroscopy (EIS) of a conventional MEA and an MEA with a multiscale membrane at 0.6 V compared with RHE. (Inverse triangles represent raw data and the solid line represents the fitted data.)

To quantitatively explain the effect of reduced membrane resistance and increased active surface area, both electrochemical impedance spectroscopy^39^ and cyclic voltammetry (CV)^40^ were carried out (Fig. 5; Supplementary Table 3; Supplementary Methods). The enhanced ECSA and Pt utilization are presented in Supplementary Table 2. From the electrochemical impedance spectroscopy analysis, we found relatively low ohmic resistance in the case of the constructed single cell (*in situ*) with multiscale Nafion membrane and the enhancement of ECSA was also observed from the CV test. The ECSA for our multiscale membrane was as much as 1.2-fold compared with that of the flat, which was not proportionally matched with the increment of geometrical surface area (1.96-fold than that of the flat one). This is mainly due to the fact that the only Pt/C catalysts that contacts onto the Nafion membrane affects the enhancement in ECSA and the Pt/C agglomerates that do not contact the membrane surface would not contribute to the increase of ECSA. Therefore, for ambient case, ∼20% increase of ECSA and 16% reduction in membrane resistance for our multilevel multiscale case well explain ∼40% increase of maximum power density. For the pressurized case, it is known that additional pressure induces enhanced transport of oxygen to the reaction sites in the cathodic active layer^41^. Hence, more active sites are available and contribute to the enhancement of performance for both flat and multiscale membranes and, therefore, the effect of increased ECSA for the multiscale case would become smaller. In this case, the reduced membrane resistance of the multiscale case would play a main role in enhancing the performance, which could explain the smaller performance enhancement for the pressurized case compared with the ambient pressure case.

We also performed accelerated durability testing (ADT) to confirm the robustness of our multiscale MEA and address if there is a risk of chemical short between the anode and cathode. The ADTs were conducted using CV method in the potential range of 0.05–1.20 V versus RHE and with a scan rate of 100 mV s^−1^ at room temperature for 5,000 cycles with fully humidified H_2_/N_2_ gases supplied to anode and cathode, respectively (in accordance with DOE condition^42^). After ADT tests, the maximum power density of multiscale MEA was found to be still higher than that of the flat membrane case by ∼22.6% (∼29.0%) in the case of H_2_/O_2_ (H_2_/air) conditions under ambient pressure and ∼9.5% (∼16.1%) in the case of H_2_/O_2_ (H_2_/air) conditions under outlet pressure of 150 kPa. There was no breakdown of the system even with long-term electrochemical stress. However, after ADT test, performances of both MEA cases with flat and multiscale membranes were decreased as shown in Supplementary Fig. 16, due to the degradation of Pt electrocatalyst and increased interfacial resistance between the Nafion membrane and the cathode catalyst layer. To examine the cause, we measured ECSAs of MEAs of the multiscale patterned and flat membranes before and after ADT tests (Supplementary Fig. 17) and found that the ECSAs in both cases decreased by ∼37%.

Summarizing, we report a novel multiplex lithography that can manufacture multilevel multiscale architectures. To do this, we utilized oxygen inhibition effects on ultraviolet-curable resin by controlling the spatial distribution of oxygen concentration in the resin. This deliberate control of spatial oxygen concentration enables to induce different curing contrasts in different parts of the resin leading to MCBs. Three different regions having different oxygen concentrations are developed in the resin using highly permeable PDMS stamp and blanket. High-concentration region allows us to imprinting, while intermediate region provides bonding and low-concentration region as a frame work, which explains how LEGO-like integration of micrometre membranes in our method is possible together with versatile multiscale pattern formation. The multiplex lithography was applied to make multiscale Nafion membrane, which not only showed higher fuel cell performance caused by the combination of the effects of lower membrane resistance and larger ECSA than the conventional flat membrane, but also provided the robustness of the membrane.

## Methods

### Fabrication of the ultraviolet-cured MCBs

A small amount of hydrophilic resin (PUA311; Minuta Tech., Osan-si, Gyunggi-do, Korea) was dispensed dropwise onto a patterned PDMS mould (see more details in Supplementary Methods), and a flat PDMS upper mould was uniformly placed onto the patterned PDMS mould. Then, the sandwich assembly was exposed to ultraviolet light (<3 min, *λ*=250–400 nm) under an applied pressure (25 g cm^−2^ ∼1 kg cm^−2^) after achieving conformal contact. After the removal of the upper and lower PDMS moulds, a flexible, free-standing MCB via the micro-ebb-tide phenomena was obtained.

### Multiscale architectures via imprinting and bonding process

A prepared brick was uniformly placed onto a nanopatterned PUA311 mould contacting the most cured face (*α*-phase) of the membrane, and the nanopatterned PDMS mould was placed onto the less cured face (*α*′-phase) of the membrane. Then, the sandwich assembly was exposed to ultraviolet light (>3 min, *λ*=250–400 nm) with hydraulic pressure (6–8 kg cm^−2^) in a vacuum chamber (5 × 10^−2^ torr) after forming conformal contact. After removal of the PDMS moulds, a multiscale two-level PUA311 hole pattern array was obtained via a simultaneous bonding and imprinting process. The same process was conducted with different microsized membranes to obtain the trilevel PUA311 hole patterned master. After preparation of the master, a mixture of base and curing agents (10:1 w/w) of Sylgard 184 PDMS elastomer was poured onto the patterned masters and cured at 70 °C for 2 h. The cured PDMS replica was peeled off from the master and cut before use.

### Preparation of the multiscale Nafion membrane

The Nafion 212 membrane (Dupont, Wilmington, Delaware, United States) was uniformly placed onto an as-prepared multiscale PDMS mould and glass substrate. Then, the sandwich assembly was imprinted under hydraulic pressure (10–20 kg cm^−2^) and temperature (∼120 °C) for 30 min. After cooling down to room temperature, the patterned Nafion membrane was peeled off the PDMS mould and kept in a deionized water container for ∼12 h.

### MEA preparation

A catalyst slurries for the anode and cathode catalyst layer were fabricated by mixing 40 wt.% Pt/C (Johnson Matthey, London, United Kingdom), Nafion ionomer solution and 2-propanol (Sigma Aldrich, St. Louis, Missouri, United States). Multiscale and flat Nafion membranes were used after the pretreatment. They were boiled in 3% hydrogen peroxide solution and rinsed in deionized water. Thereafter, the membranes were soaked in 0.5 M H_2_SO_4_ and washed again in deionized water. Each procedure in the solutions was performed at 80 °C for 1 h. The prepared catalyst slurries were sprayed onto the anode and cathode parts of the Nafion membrane. The Pt loadings were 0.12 mg cm^−2^ in both the anodes and cathodes of the MEAs. Pt loading was measured from the weight difference before and after spraying Pt/C catalyst ink onto PET film (*W*_Pt_=*W*_(Pt+PET)_−*W*_PET_). As shown below, we made a calibration curve for the deposited amount of Pt as a function of a catalyst ink volume from which we chose the loading of 0.12 mg cm^−2^ based on the DOE condition. The catalyst-coated membranes were dried at room temperature for 12 h and situated between the anode and cathode gas diffusion layers (SGL Carbon, Wiesbaden, Germany) without a hot press process. The active geometric areas of the MEAs were 5.0 cm^2^.

### Physical analysis

Fourier transform infrared (FTIR) spectroscopy spectra were obtained using Vertex 70 with FTIR spectrometer (BRUCKER) to monitor the degree of photopolymerization of PUA as a function of the ultraviolet exposure time. The curing contrast or conversion ratio was calculated as


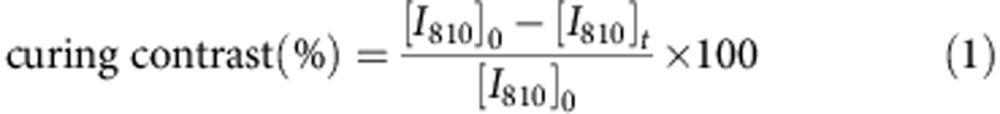


where [*I*_810_]_0_ and [*I*_810_]_*t*_ are the signal intensities at time=0 and *t*, respectively.

## Additional information

**How to cite this article:** Cho, H. *et al*. Multiplex lithography for multilevel multiscale architectures and its application to polymer electrolyte membrane fuel cell. *Nat. Commun.* 6:8484 doi: 10.1038/ncomms9484 (2015).

## Supplementary Material

Supplementary Information Supplementary Figures 1-17, Supplementary Tables 1-3, Supplementary Notes 1-3 and Supplementary Methods

Supplementary Movie 1 Movement of the PUA micro ebb tides toward the neighboring PUA brick due to the low affinity for the hydrophobic PDMS surface.

Supplementary Movie 2 Dewetting of the PUA droplet on the disintegrated flat PDMS layer after short UV exposure of the sandwich-like PDMS assembly.
