# Supplementary material for: Multiplex lithography for multilevel multiscale architectures and its application to polymer electrolyte membrane fuel cell
Source: Nat Commun. 2015 Sep 28;6:8484. doi: 10.1038/ncomms9484 (PMC4598841; doi:10.1038/ncomms9484)
Supplement: Supplementary Information — Supplementary Figures 1-17, Supplementary Tables 1-3, Supplementary Notes 1-3 and Supplementary Methods [file ncomms9484-s1.pdf]

Supplementary Information

Supplementary Figures

a

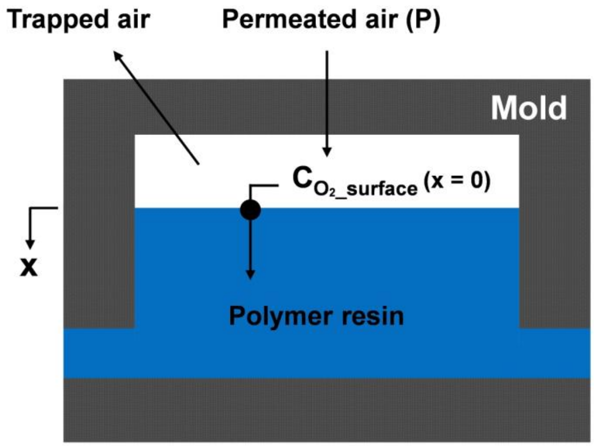

$$-\frac{dC_{O_2}}{dt} = -D_{O_2/polymer}\frac{d^2C_{O_2}}{dx^2} + kC_{O_2} - P,\ \text{(At steady-state, } \frac{dC_{O_2}}{dt} = 0)$$

$$\frac{C_{O_2}(x)}{C_{O_2\_surface}} = \cosh mx - \tanh mL * \sinh mx\,, \qquad m = \left(\frac{k}{D_{O_2/polymer}}\right)^{1/2}$$

b

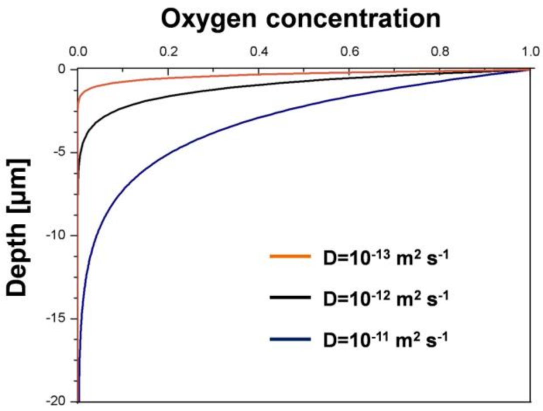

Supplementary Figure 1. a, Schematic illustration for the oxygen permeation, diffusion and chemical reaction in the polymer resin and the corresponding theoretical model under a permeable boundary. **b,** Computational plot of the oxygen concentration in accordance with the three different diffusivities of oxygen into the PUA resin (D=10^{-13} – 10^{-12} m^2 s^{-1}).

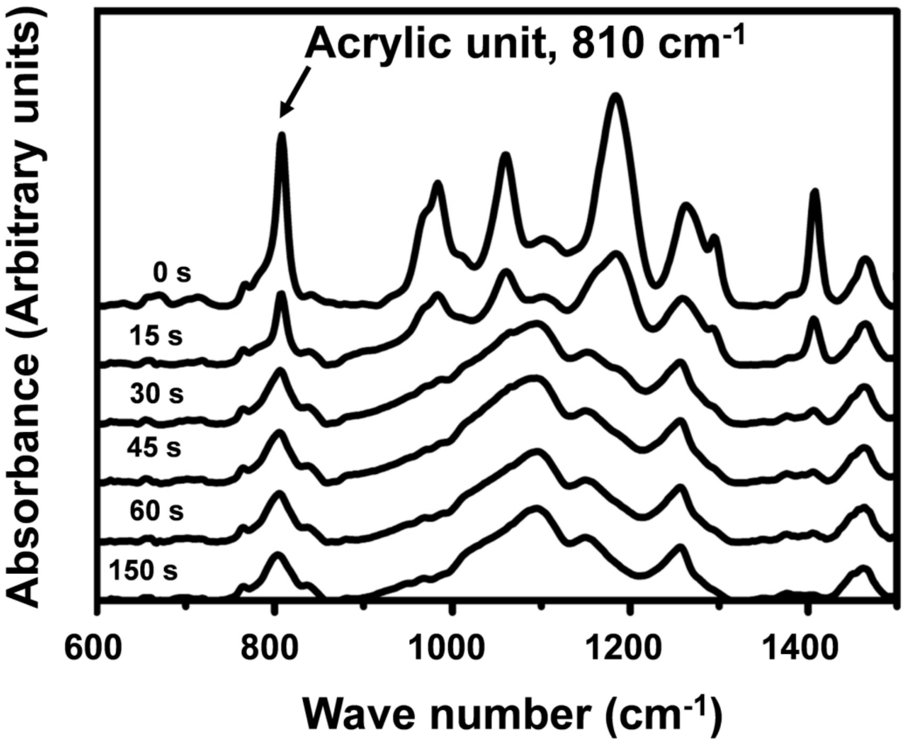

Supplementary Figure 2. **Quantitative analysis of the degree of multiplex curing by FT-IR spectroscopy.** FT-IR spectra with various UV exposure times (0, 15, 30, 45, 60, and 150 s) to quantitatively discuss the scavenging effects of oxygen under the permeable PDMS blanket.

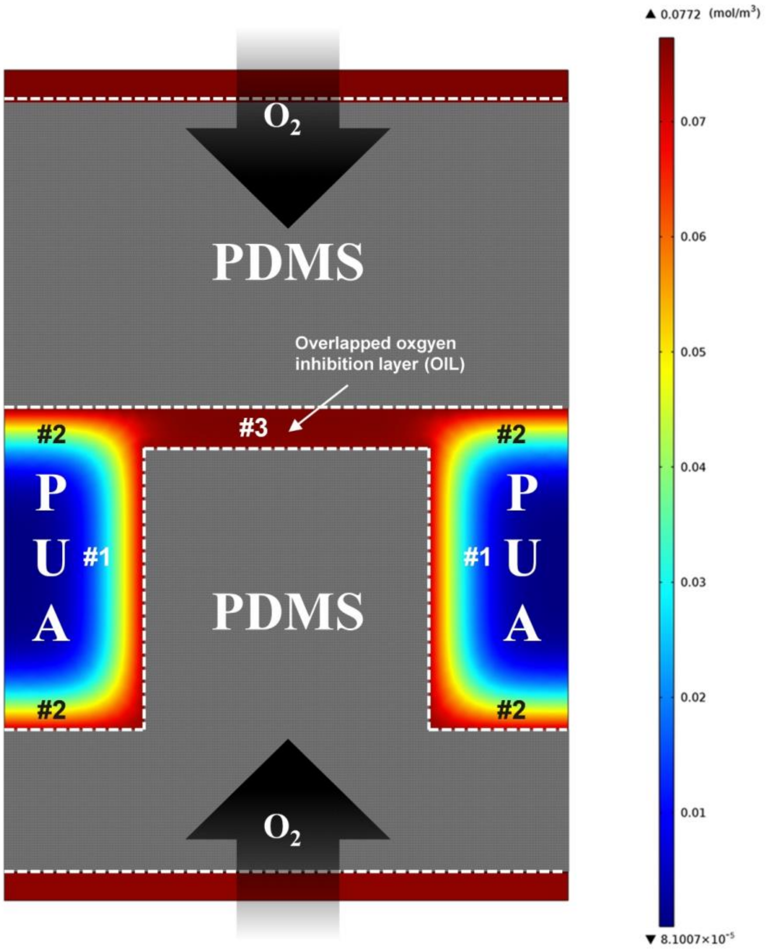

Supplementary Figure 3. Simulation study related to oxygen diffusion model for the generation of a MCB.

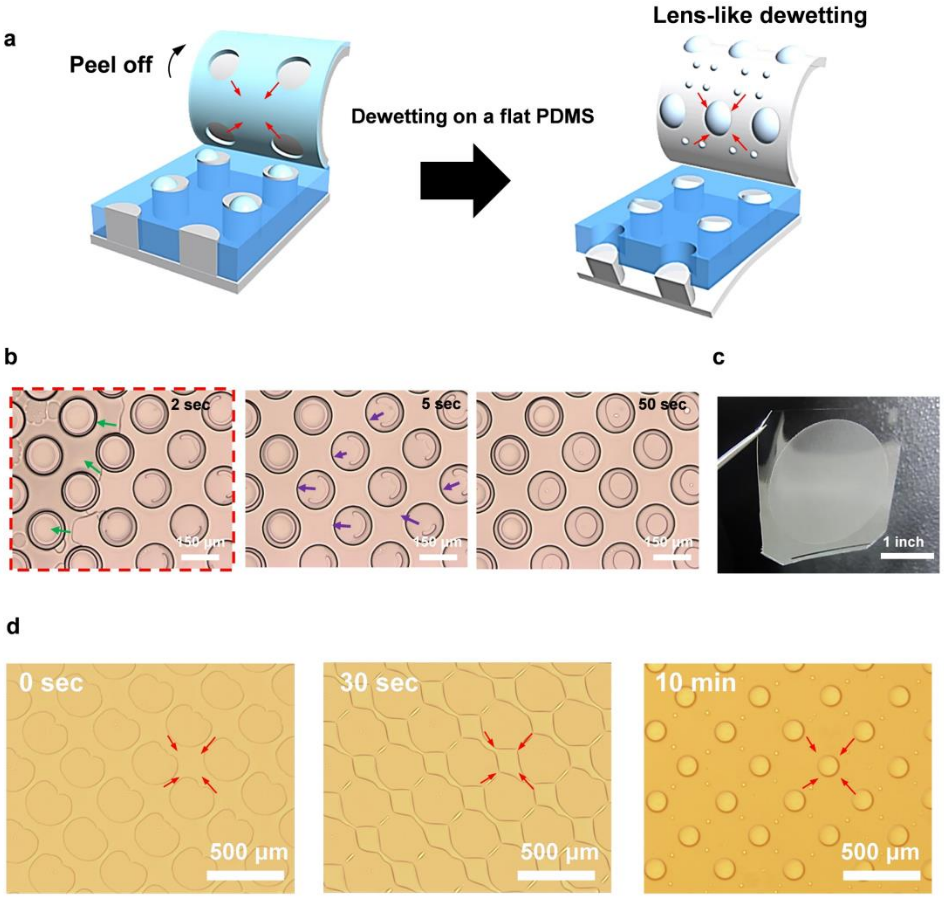

Supplementary Figure 4. Direct dewetting of insufficiently cured PUA resin on a disassembled flat PDMS layer. a, Schematic illustration displays an array of lens-like hemispherical dewetting of PUA resin after the peel-off of the flat PDMS, where affinity differences between mould and resin result in the quietly slow dewetting of detached PUA layer over time. **b,** Microscopic images of the real-time monitoring of micro ebb tides. The low affinity of viscoelastic PUA resin to PDMS pillars yields the spontaneous coating of partially cured resin on the top surface of the brick. **c,** Digital image of the MCB produced as a unit part for LEGO®-like stacking. **d,** Microscopic images demonstrating the dewetting phenomenon, which minimizes the surface area during a formation of individual hemispherical droplets. Scale bar: 500 μm.

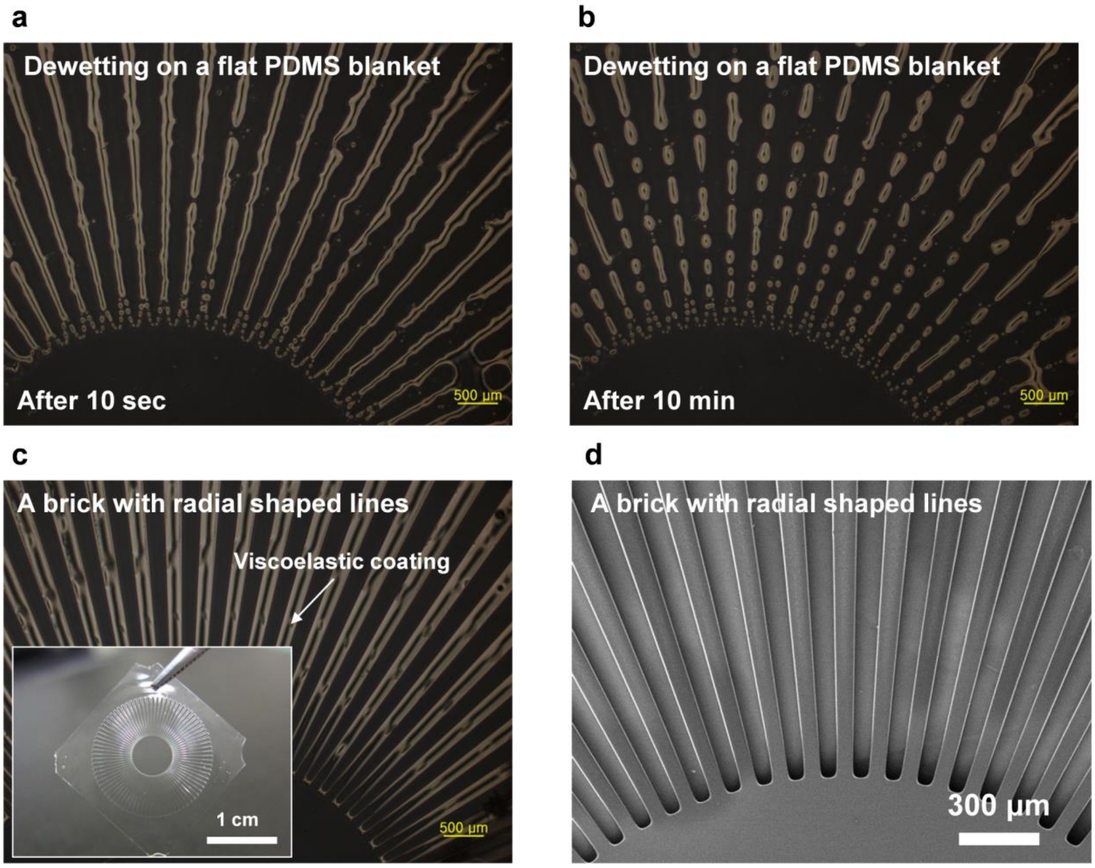

Supplementary Figure 5. Experimental results for a radial-shaped membrane after the short 1st UV exposure process. a,b,c, The microscopic images display the dewetted PUA droplet over time onto the disintegrated flat PDMS blanket (**a, b**) as well as the free-standing radial-shaped membrane (**c**). The microscopic image shows the viscoelastic resin as the white-coloured regions along the structures, whereas the same area was not observed in the SEM image (**d**). Scale bar: 500 µm.

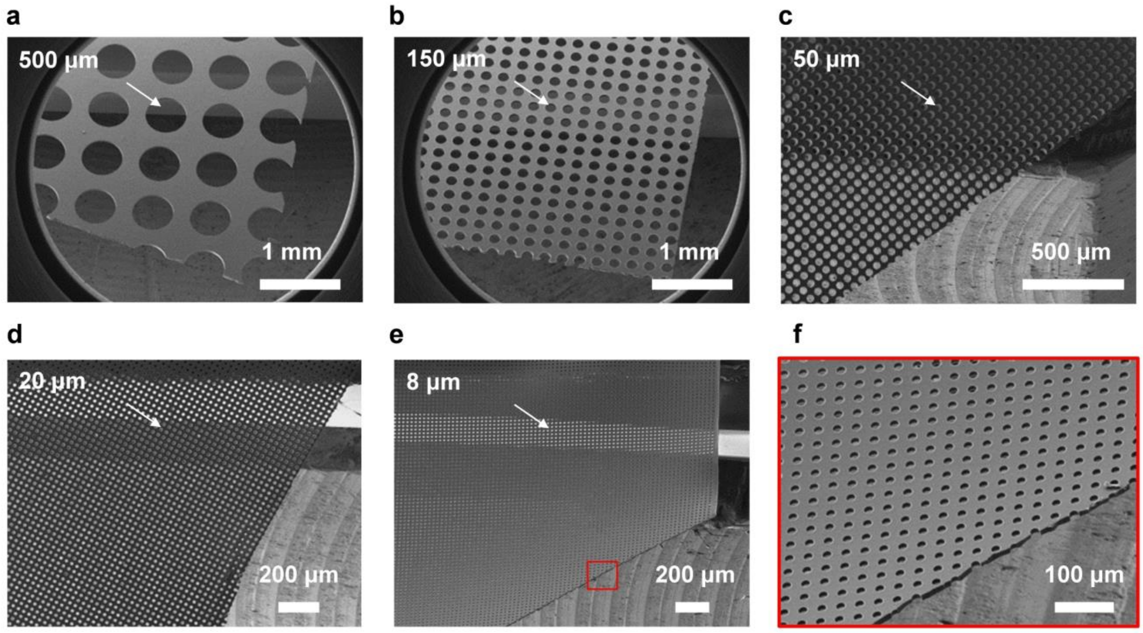

Supplementary Figure 6. SEM images of the obtained MCBs with various micro apertures. The MCBs were fabricated after the 1st curing process and peel-off process. From this method, we can yield various MCBs with designed micro apertures. 500-μm pores (**a**), 150-μm pores (**b**), 50-μm pores (**c**), 20-μm pores (**d**) and 8-μm pores (**e**, **f**).

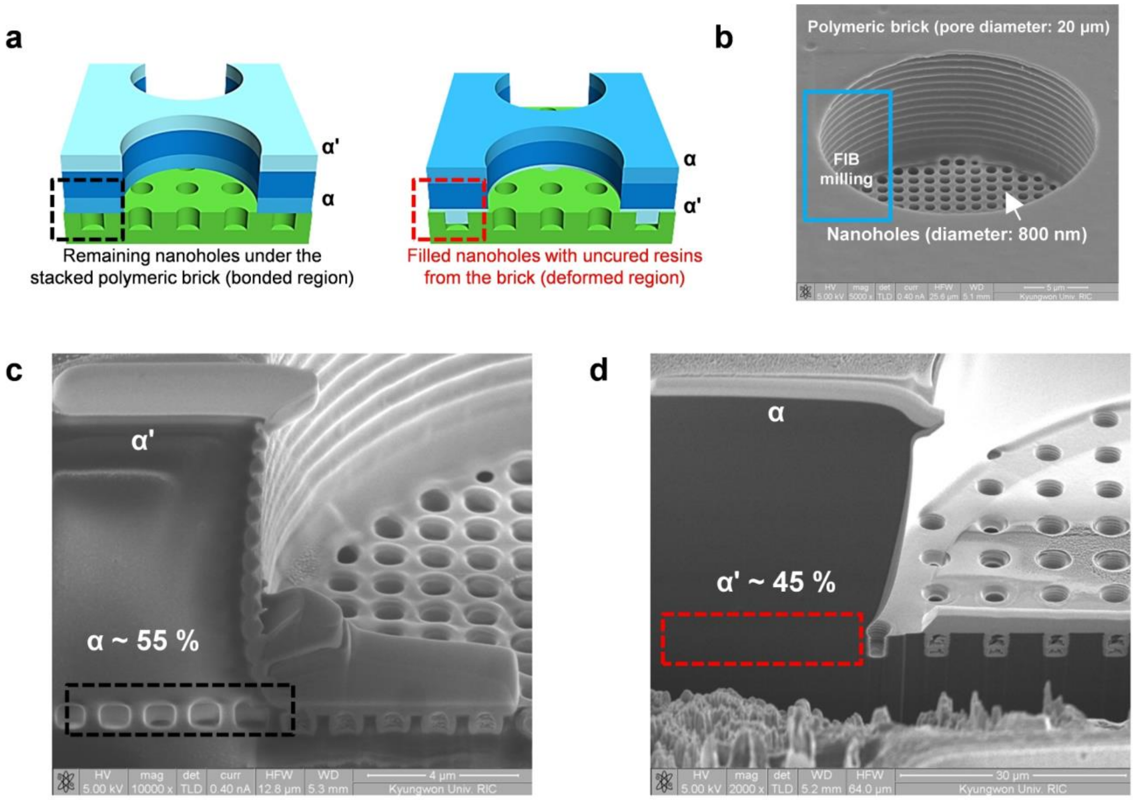

Supplementary Figure 7. a, Schematic illustration of the vertical stacking of MCBs onto the patterned substrate with high curing contrast of β showing the surficial bonding and structural interconnection according to the facial selections. **b,c,d,** SEM images of the stacked bricks (**b**) and the connected interface revealed by FIB milling (**c,d**). To analyze the cross-sectional profile, FIB studies were carried out to observe the bonded interfaces under the connected moulds at a fixed ratio with a diameter of 25 (= d_{Top}/d_{Bottom}, d_{Top} = 20, 100 μm, d_{Bottom} = 0.8, 4 μm, respectively).

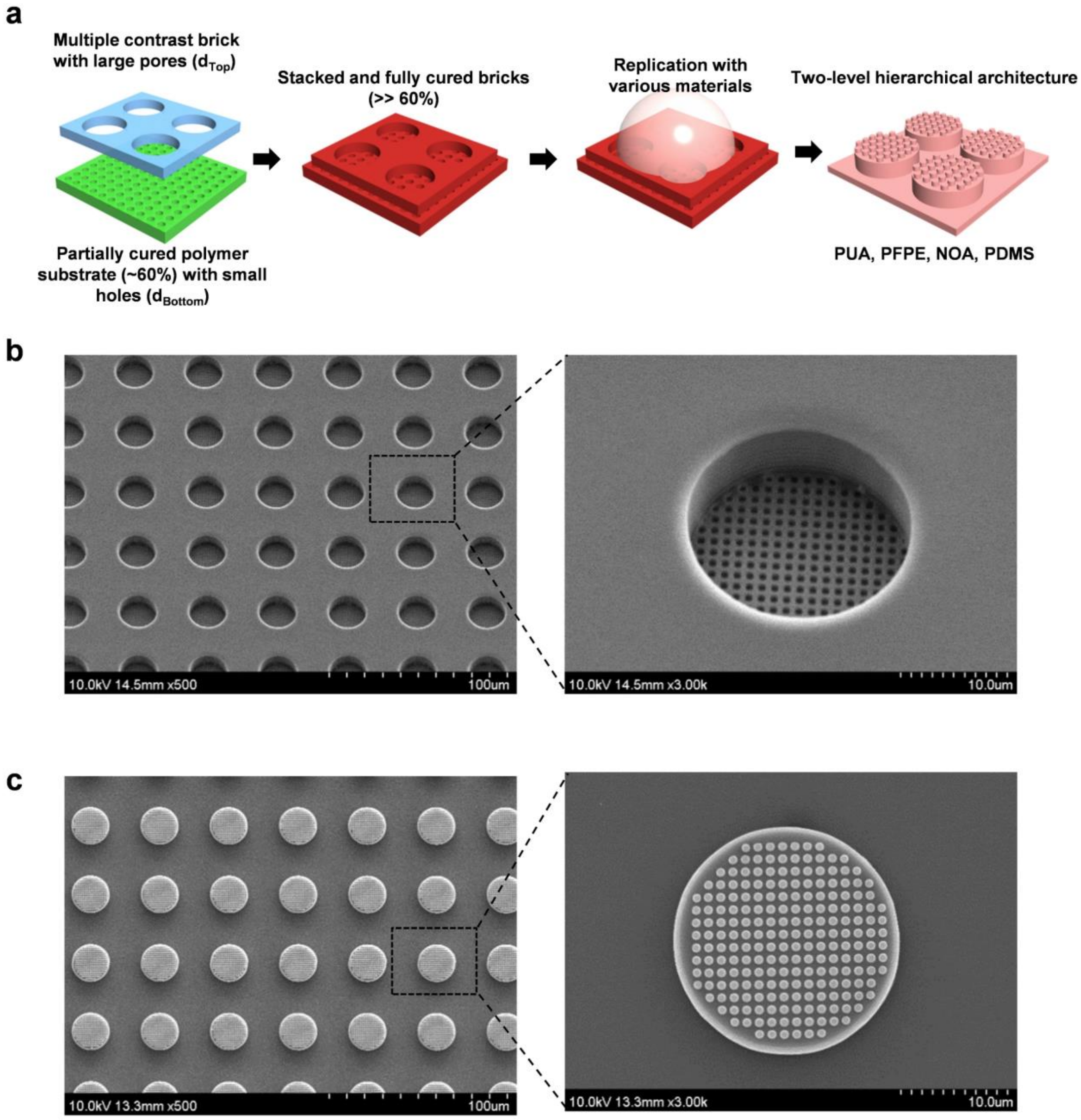

Supplementary Figure 8. Fabrication results for the hierarchical structure by the vertical stacking process. a, Schematic illustration of the stacking MCB onto the patterned substrate and the replica moulding to obtain two-level hierarchical structures from soft polymers. **b,** Tilted SEM images of the bonded structure after stacking and bonding. **c,** SEM images of the hierarchical structures generated after the replication process.

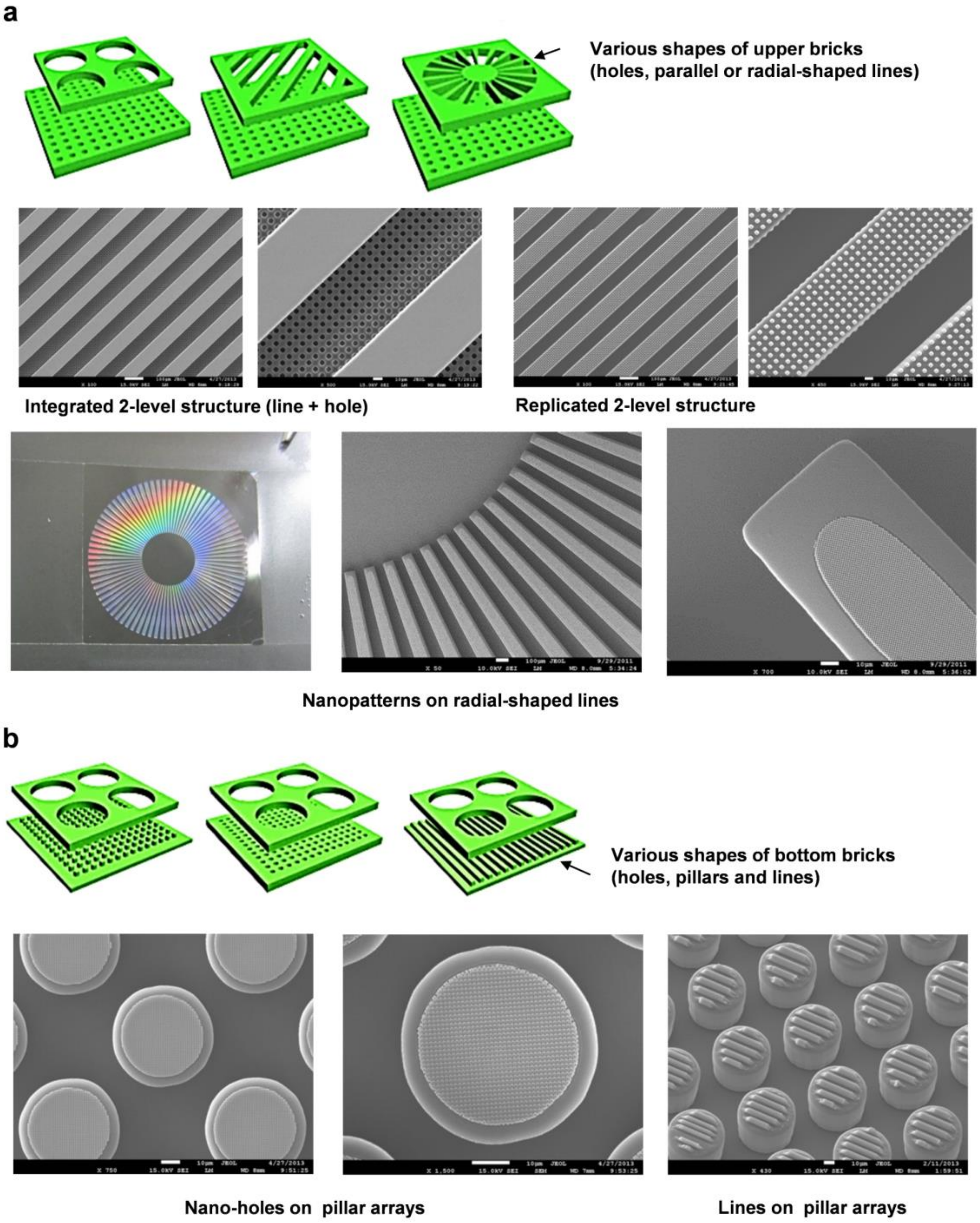

Supplementary Figure 9. Direct fabrication for hierarchical structures by performing vertical stacking process with combinations of various bricks. a, Schematic illustration for variations in the upper brick (holes, parallel or radially shaped lines) and corresponding microscopic and SEM images. **b,** Schematic illustration for variations in the bottom brick (holes, pillars and lines) and the corresponding SEM images.

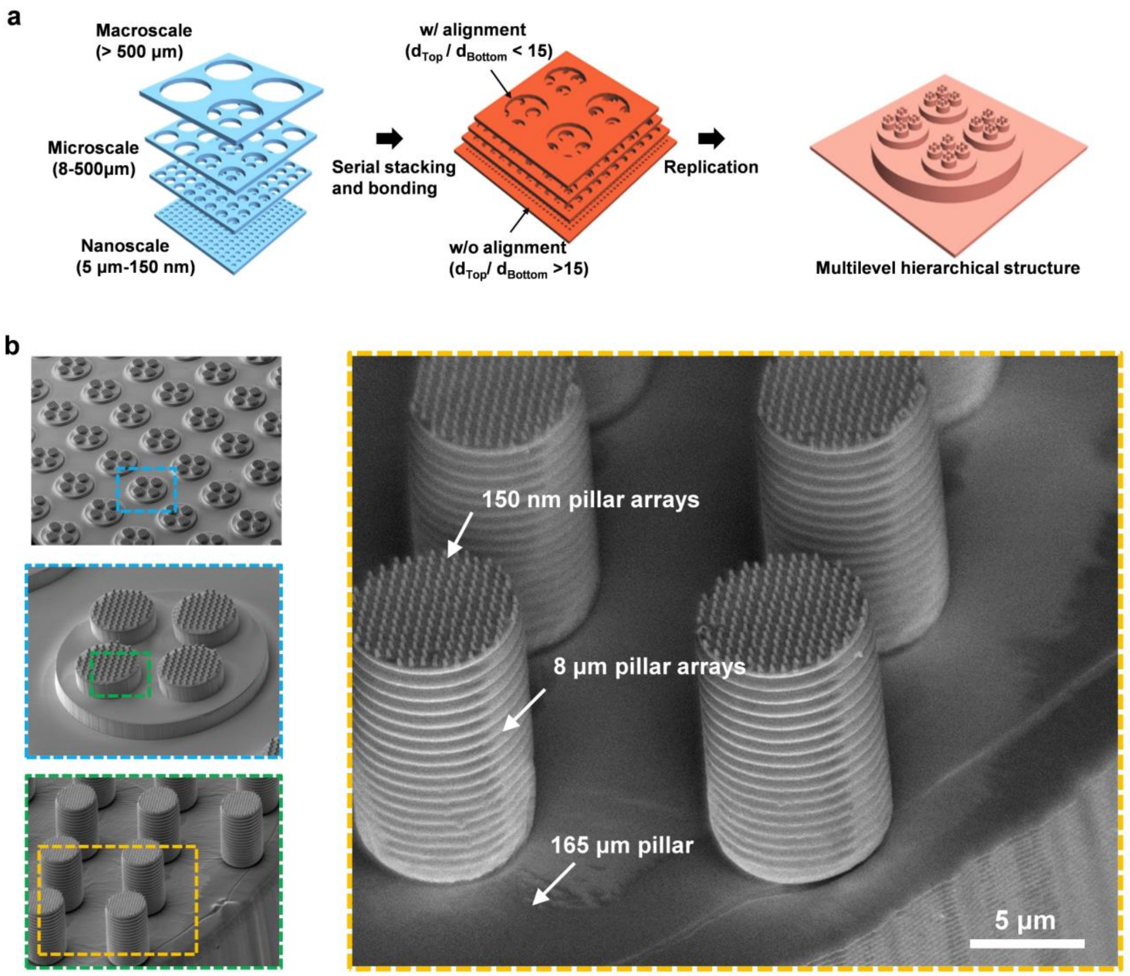

Supplementary Figure 10. Facile fabrication of multilevel, hierarchical structures of up to four levels from soft polymers. a, Schematic illustration for the fabrication of a four-level polymeric architecture via the multi-stacking process with MCBs. **b,** Multiscale perspective for the obtained four-level architecture with various pillars of increasing sizes (150 nm/8 μm/165 μm/500 μm).

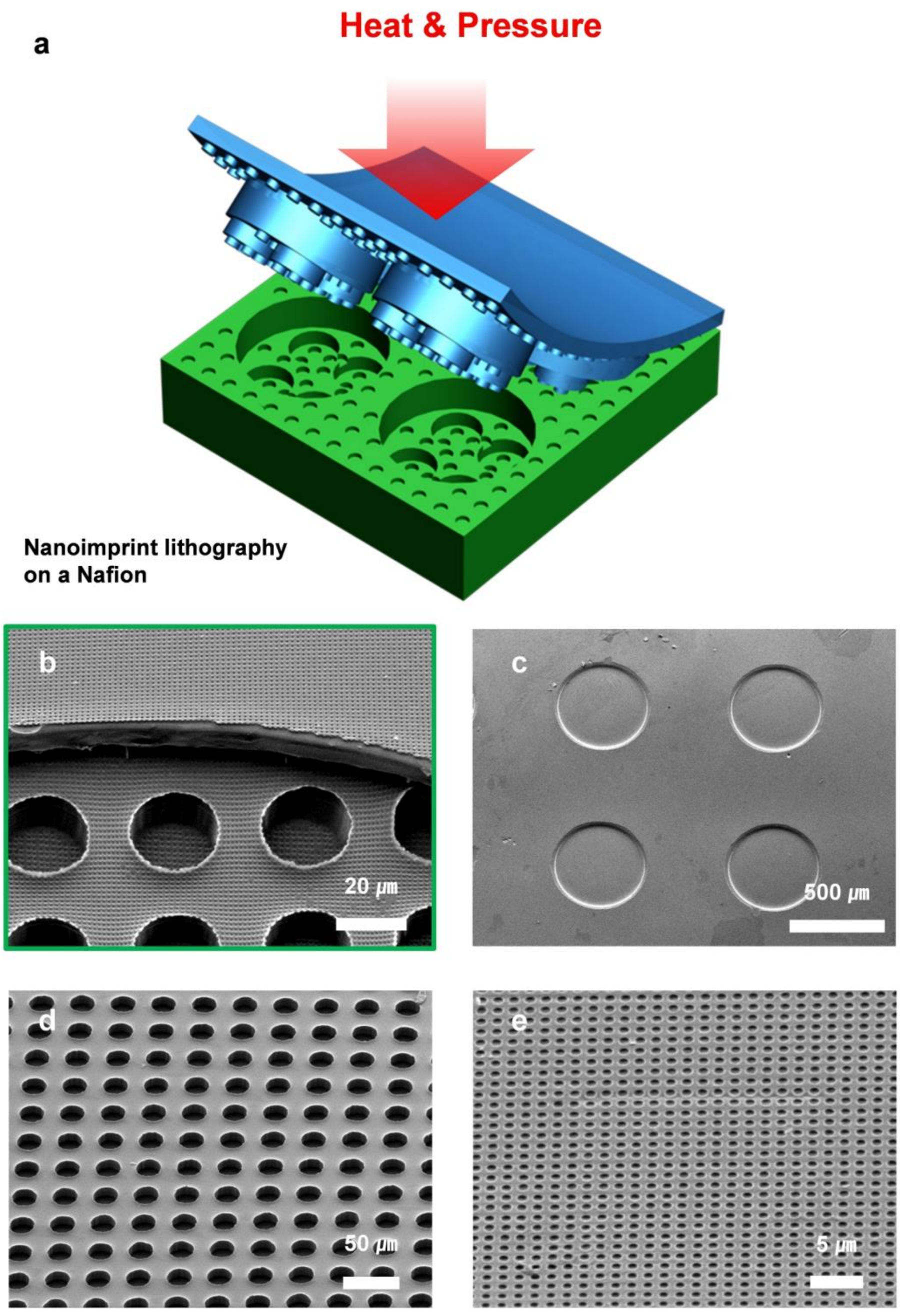

Supplementary Figure 11. a, Schematic illustration of the thermal imprinting process onto a Nafion 212 membrane. **b,c,d,e,** SEM images of the patterned Nafion membrane after the imprinting process with the pattern dimension of multiscale (**b**), 500 μm (**c**), 20 μm (**d**), and 800 nm (**e**).

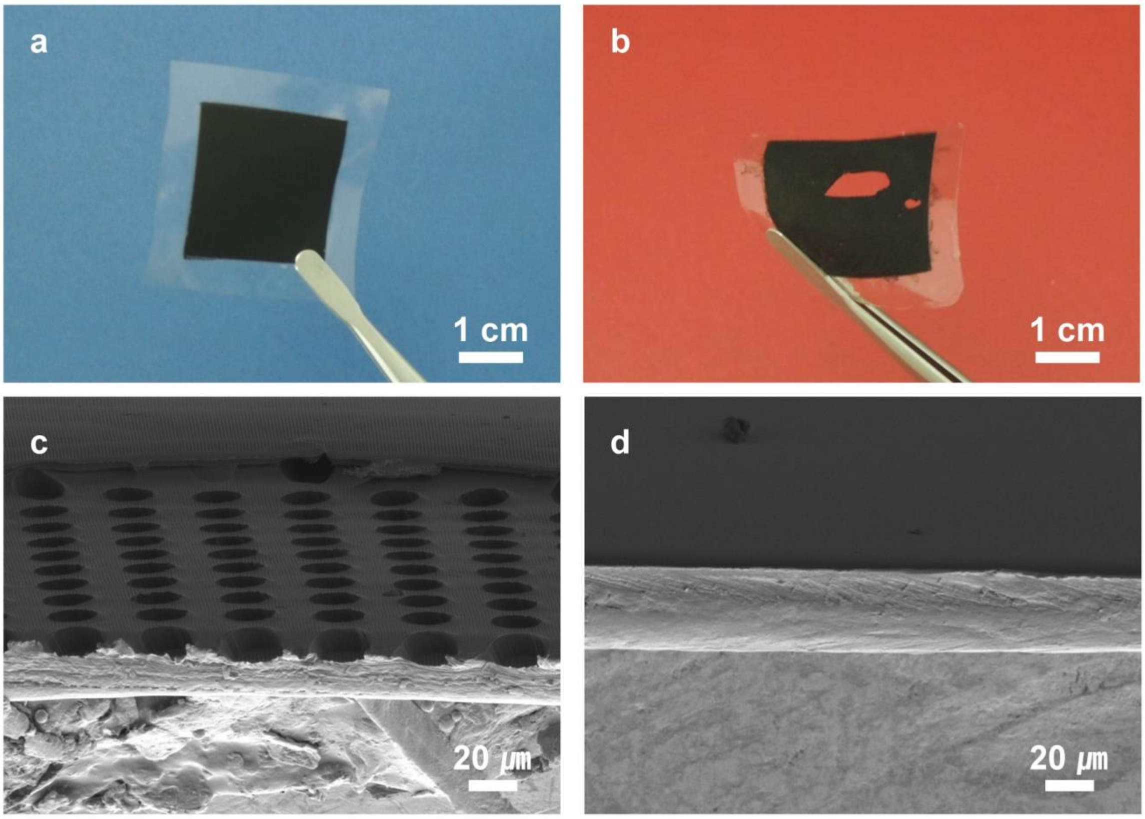

Supplementary Figure 12. Comparison of the processability when prepared by spraying the mixture of Pt/C, Nafion ionomer and 2-propanol onto both sides of the membrane. Multiscale Nafion membrane (**a**) and thin Nafion membrane (thickness < 30 μm) (**b**) and cross-sectional SEM images of each membrane (**c, d**).

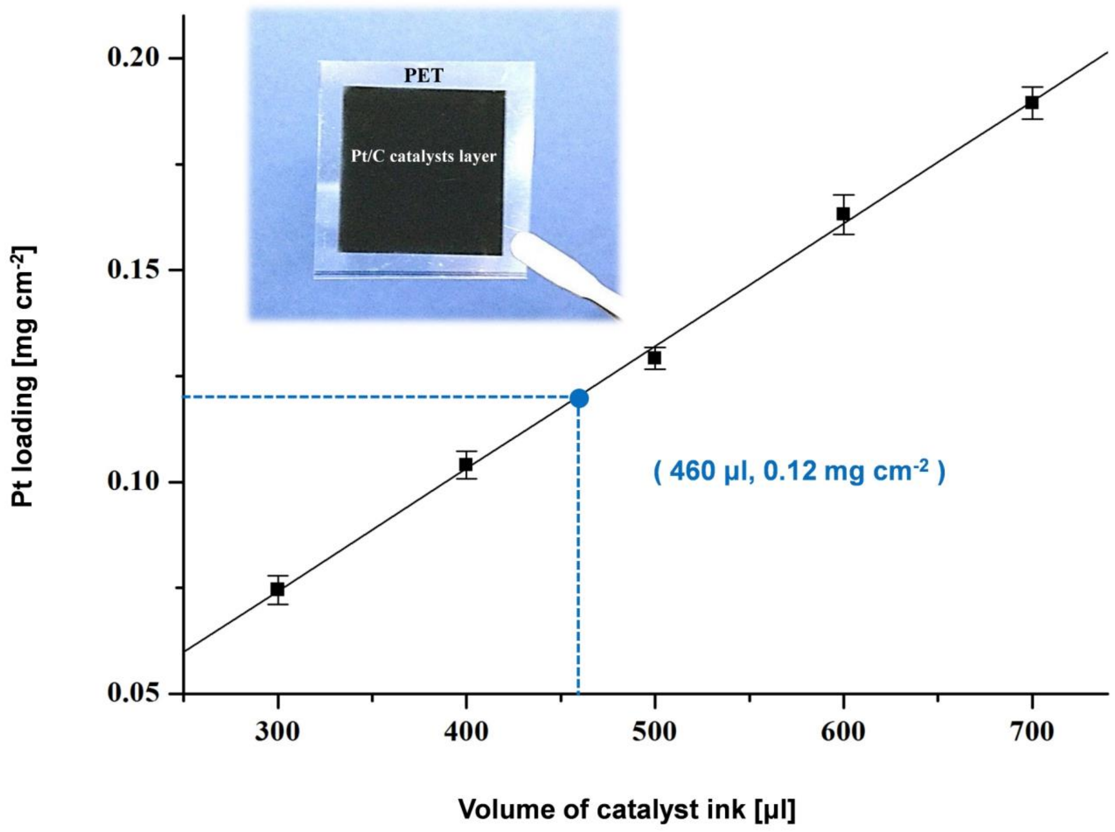

Supplementary Figure 13. Measured Pt loading depending on the volume of Pt/C catalyst ink.

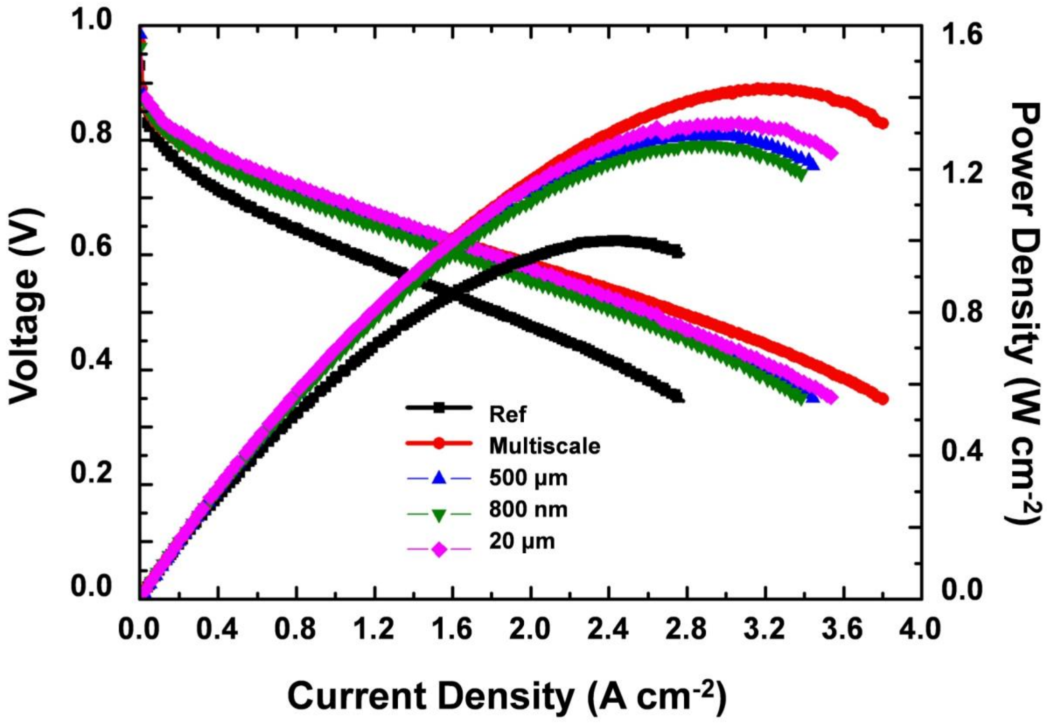

Supplementary Figure 14. Single-cell performance of the MEAs with various pattern dimensions in the conditions of H_2/O_2 under ambient pressure.

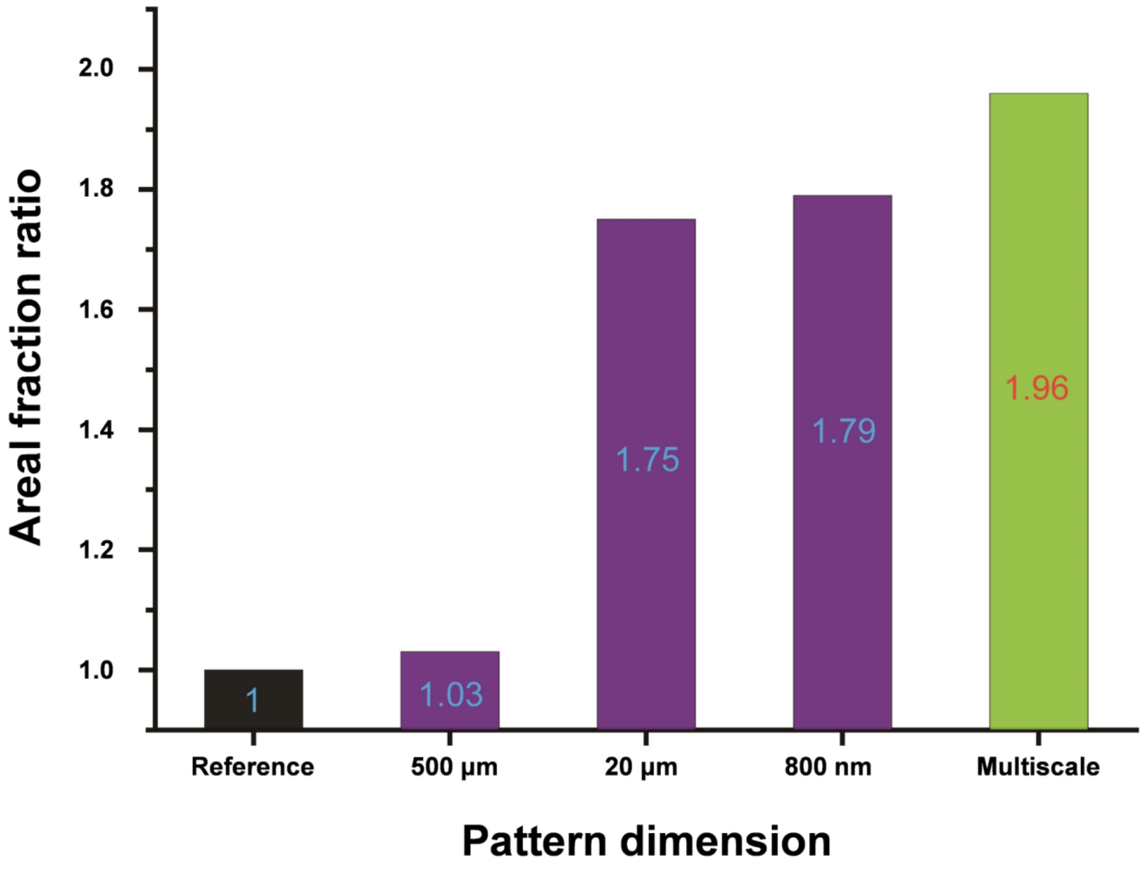

Supplementary Figure 15. Increased surface area ratio compared to a flat surface with variations in the pattern dimensions.

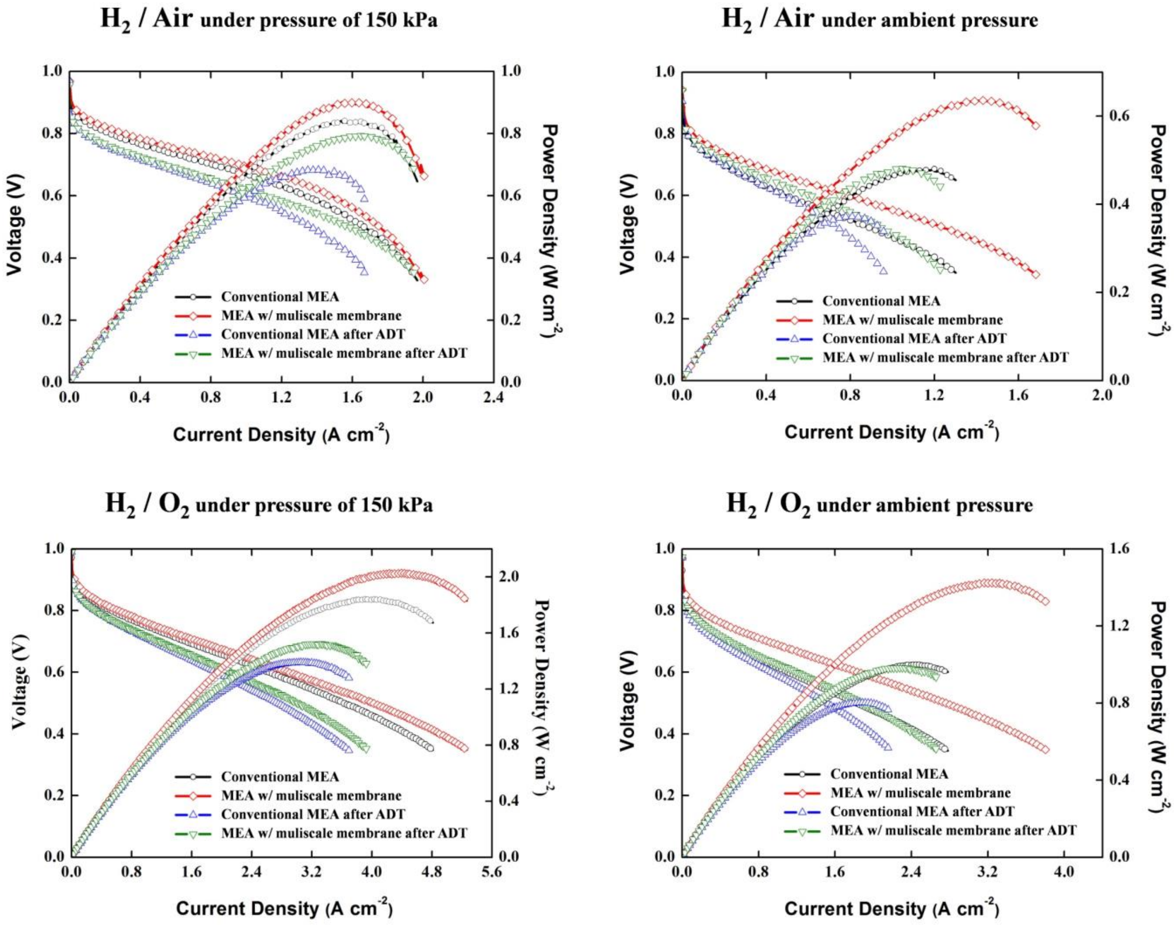

Supplementary Figure 16. Polarization curves of MEAs before and after accelerated durability test (ADT).

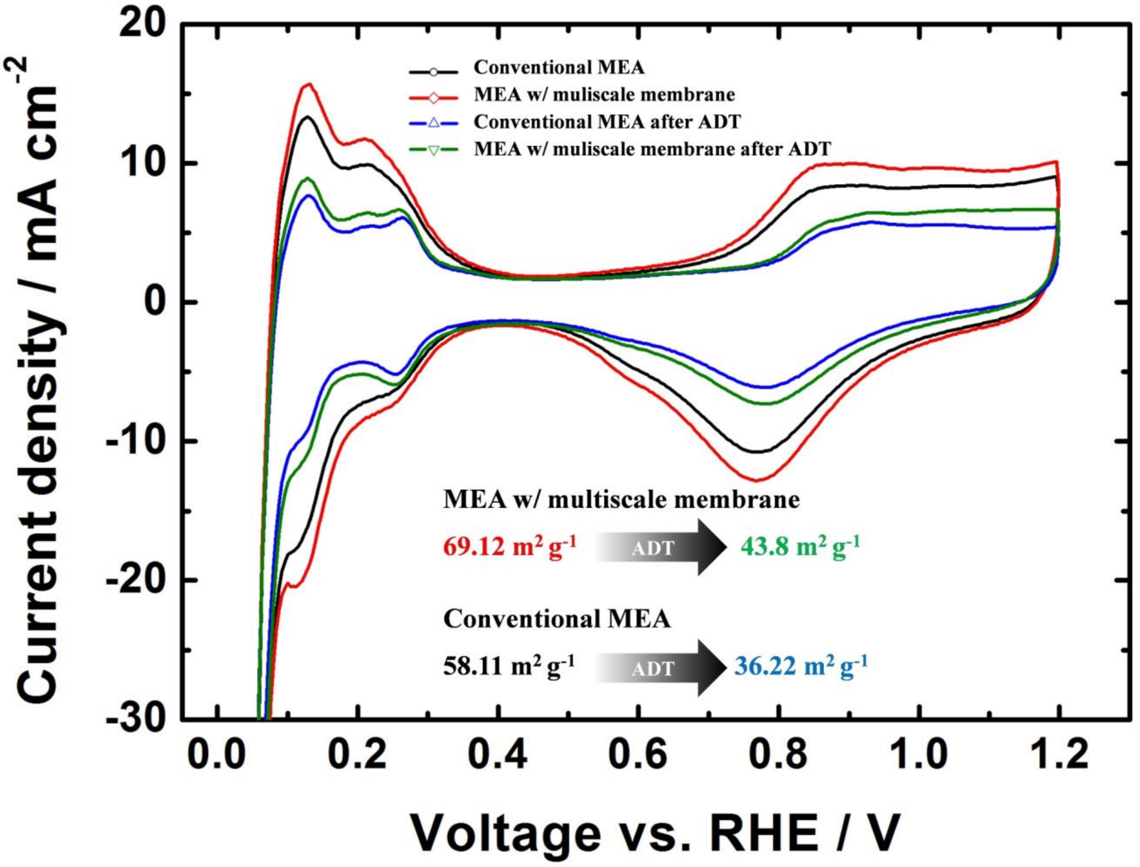

Supplementary Figure 17. Cyclic voltammograms (CV) of MEAs before and after accelerated durability test (ADT).

Supplementary Tables

Supplementary Table 1. Physical property of Nafion membrane		
	Tensile strength, max. [MPa]	*Elongation to break, %*
**Multiscale Nafion**	**27.38[a] (± 1.45)**	**306[a] (± 26)**
**Nafion 212**	**31.11[a] (± 2.49)<br>32[b]**	**300[a] (± 18)<br>343[b]**
**Nafion 211**	**23.55[a] (± 1.92)<br>23[b]**	**237[a] (± 17)<br>252[b]**

[a] **Measured values through strain tension tests .**
[b] **General properties of Nafion® PFSA Membrane presented by Dupont™**
Please, refer to www.fuelcellmarkets.com/content/images/articles/nae201.pdf

Supplementary Table 1. Physical property of Nafion membrane

Supplementary Table 2. Physical and electrochemical properties of samples

Samples	Pt loading (mg cm^{-2})	Average Pt particle size (nm)	Geometric Surface Area (m^2 g^{-1})	Electrochemical Active Surface Area (m^2 g^{-1})	Pt utilization (%)	Maximum power density (W cm^{-2})	Current density @ 0.6 V (A cm^{-2})	Power density @ 0.6 V (W cm^{-2})	Current density @ 0.8 V (A cm^{-2})	Power density @ 0.8 V (W cm^{-2})
Conventional MEA	0.12	3.00[a]	93.46[a]	58.11	62.18	0.4786[b] (1.000)	0.4906[b] (1.1104)	0.2954[b] (0.6662)	0.0202[b] (0.09)	0.0162[b] (0.072)
						0.8408[c] (1.845)	1.3272[c] (2.634)	0.799[c] (1.5804)	0.2186[c] (0.528)	0.1748[c] (0.4224)
MEA w/ multiscale membrane				69.12	73.96	0.6356[b] (1.423)	0.7810[b] (1.842)	0.4702[b] (1.1052)	0.0508[b] (0.1906)	0.0408[b] (0.1524)
						0.9000[c] (2.026)	1.4672[c] (2.894)	0.8818[c] (1.7364)	0.3086[c] (0.618)	0.2468[c] (0.4944)

[a] Reference # 35

[b] Cell operation under ambient pressure with H_2/air (H_2/O_2).

[c] Cell operation under pressure of 150 kPa with H_2/air (H_2/O_2).

Supplementary Table 2. Physical and electrochemical properties of the samples.

Supplementary Table 3. EIS fitted data					
	R_{anode}	$CPE_{anode\text{-}T}$	$R_{membrane}$	$R_{cathode}$	$CPE_{cathode\text{-}T}$
**MEA w/ multiscale membrane**	**0.00250**	**0.0520**	**0.0131** *(79.4 %)*	**0.0354** *(80.5 %)*	**0.0660** *(129.4 %)*
**Conventional MEA**	**0.00250**	**0.0510**	**0.0165** *(100 %)*	**0.0440** *(100 %)*	**0.0510** *(100 %)*

Supplementary Table 3. EIS fitted data.

Supplementary Notes

Supplementary Note 1:

Observation of the micro ebb tides after the 1st UV curing

The microscopic images in Supplementary Fig. S4b are monitoring the events for the micro ebb tides that act as an automatic viscoelastic coating on a neighboring PUA brick (see also Supplementary Movie S1). Micro ebb tides of partially cured resin were observed and utilized to form multiple contrast bricks (MCBs) with a viscoelastic coating that was shaped into nanopatterns and connections during multiple UV exposures. As an initial step, a long wave occurs rapidly by the surface tension of the uncured PUA resin (green arrows). Then, the individual viscoelastic droplet starts to move as a result of affinity mismatching (purple arrows). After the dewetting, we could obtain a MCB as a basic brick for the vertical stacking process by peeling off the layer from the dewetting surface. Supplementary Fig. S4c is a representative digital image of the MCB with 150-μm apertures over a large area of up to 2 inches. This type of constant, slow dewetting lasted for several minutes (> 1 min), where similar dewetting is also detectable on the disintegrated flat PDMS layer (Supplementary Fig. S4d and Movie S2).

Supplementary Note 2:

Vertical stacking of MCBs and observation of the interconnections

We vertically stacked the obtained MCB on a holey polymeric substrate under a surface selection (c ~ α and α') to elucidate the role of deformation of the viscoelastic coating on the MCB (Supplementary Fig. S8-9). According to the calculation based on the relative peak height of the FT-IR spectra with respect to the uncured resin, the curing contrast of the top surface of MCB showed a low value (α' ~ 45%) due to the coating with viscoelastic micro ebb tides, whereas the bottom surface of MCB had a relatively high curing contrast (α ~ 55%). In the stacking experiments, the both surfaces (α and α') of MCB ensured stable connecting

results, although the underlying holey bottom substrate (PUA) was prepared with a relatively high curing contrast (c~ 60%) for good pattern fidelity at the submicron scales as well as high-resolution replications of the hierarchical polymeric structures. The scanning electron microscopy (SEM) images in Supplementary Fig. S7 show the clearly attached 20-μm holes on the underlying substrate with an array of 800-nm patterns. To match the cross-sectional profile with the calculated curing contrast, a focused ion beam (FIB) study was performed by revealing the connected interface under the stacked MCB at a fixed ratio with a diameter of 25 (= d_{Top}/d_{Bottom}, d_{Top} = 20, 100 μm, d_{Bottom} = 0.8, 4 μm, respectively), which guaranteed the proper shape of the hierarchical structure after the monolithic stacking of each MCB (Supplementary Fig. S7). When a surface with a curing contrast of α (d_{Top} = 20 μm) is selected and attached to the pattern with nanoholes (d_{Bottom}=0.8 μm), these underlying nanoholes remain clearly unfilled under a bonding interface after the monolithic integration (we termed this as "surficial bonding") (Supplementary Fig. S7c). However, for a stacking with the viscoelastic coating of α' (d_{Top} = 100 μm), even the microholes (d_{Bottom} = 4 μm) were completely filled with the resin (we termed this as "structural interconnection"), suggesting that the structural deformation from fluidity of the viscoelastic coating was sufficiently high at the interface (Supplementary Fig. S7d). The existence of top surface of MCB with low curing contrast of α' is surely important to perform the multiplex lithography and we utilized the layer for nanopatterns via a secondary nano-moulding process. We note that the bottom surface with curing contrast of α is also possible to be connected with the underlying nanopatterns; thus various combinations with bricks were demonstrated to form two-level hierarchical structures via the stacking strategies. The replication results showed a facile generation of two-level hierarchical structures from various polymers by combining two bricks under a facile manner (see Supplementary Fig. S8-9), which directly enables the rapid prototyping of multilevel hierarchical architectures through this simple vertical stacking method.

Supplementary Note 3:

Rapid prototyping of multilevel hierarchical structures via serial stacking procedures.

This monolithic integration concept was further verified by stacking various shapes of bricks under combinations of the top and bottom layers for a generation of successful multilevel architectures of up to four levels (Supplementary Fig. S10). By monolithically stacking the four bricks, which constituted a series of increasing holes in sizes (150 nm/8 μm/165 μm/500 μm), and executing facile UV-replicating processes, a four-level hierarchical architecture was precisely formed with or without the alignment of the MCBs. The four-level architecture shown in the Supplementary Fig. S10 was formed based on these design rules, providing a key idea for indeed multiplex lithography under a utilization of viscoelastic deformation of the top surface with a low curing contrast (α') and multiple exposure of UV to generate independent nano patterns on every projection on the hierarchical multilevel structures.

Supplementary Methods

Preparation of the PDMS patterned array. The hole patterned silicon masters were fabricated by photolithography and reactive ion etching. The masters were treated with a fluorinated-SAM solution (tridecafluoro-1,1,2,2-tetrahydrooctyl)-trichlorosilane:FOTCS, (Gelest Corp., Morrisville, Philadelphia, United States) diluted to 0.03 M in anhydrous heptane in an Ar chamber. After the surface treatment, the masters were annealed at 120°C for 20 min. After the preparation of the master, a mixture of base and curing agent (10:1 w/w) of Sylgard 184 PDMS elastomer (Dupont, Wilmington, Delaware, United States) was poured onto the patterned masters and cured at 70°C for 2 h. The cured PDMS moulds were peeled off from the masters and cut prior to use.

Physical analysis. Magnified optical images were observed using an optical microscope (Olympus IX70, Japan). Field emission-scanning electron microscopy (FE-SEM) was conducted using a SUPRA 55VP microscope (Carl Zeiss) to measure the morphology of the various samples used in this paper.

COMSOL simulation. The simulation study related to mechanical strength of Nafion membranes was conducted by using linear elastic material model in condition of two edges fixed and applied same stress (100 kPa) perpendicular direction onto the surface. In addition, simulation study related to oxygen distribution in the case of the generation of a MCB by slightly curing the PUA resin between two permeable PDMS layers was conducted by using diffusion model in condition of respectively specific diffusivity of the materials (D_{PDMS} ~ 3.55×10^{-9} m^2 s^{-1}, D_{PUA}~ 10^{-12} m^2 s^{-1}) and initial concentration value (c=1 in top and bottom side and c=0 in the rest).

Electrochemical measurements. Cyclic voltammograms (CVs) were obtained at 100 mV s^{-1} between 0.05 and 1.20 V to measure the electrochemical active surface (EAS) of the prepared cathode catalyst layers at room temperature. Humidified H_2 and N_2 gases were supplied to the anode and cathode, respectively, and the relative humidity (RH) was 100%. The anode-flowing H_2 gas was used as the reference and counter electrodes, and the cathode-flowing N_2 gas served as a working electrode. For the single cell performance test at 80°C, humidified H_2 and O_2 (air) gases were flowed into the anode and cathode, respectively. The stoichiometric coefficient of H_2/O_2 (air) was 2.0/9.5 (2.0), with a total outlet pressure of 150 kPa. Additionally, the RHs for the anode and cathode gases were 100%. Electrochemical impedance spectroscopy (EIS) (IM6, Zahner) of the single cells was measured at 0.6 V with an amplitude of 10 mV. The measurement was conducted in the frequency range from 0.1 Hz to 100 kHz. Other experimental conditions, such as temperature and gas humidification, were the same as for the single-cell operation at 80°C with H_2/Air. The ZView program (Scribner Associates Inc., Southern Pines, North Carolina, United States) was used to fit the EIS data, and a simple equivalent circuit was applied. The proton conductivity of Nafion membranes was measured using EIS (IM6, Zahner) fitted with four probes with an amplitude of 10 mV over the frequency range from 0.1 Hz to 3.0 MHz. The impedance was measured at a controlled humidity (90% RH) and temperature (70°C). All electrochemical measurements in this study were reported relative to a reversible hydrogen electrode (RHE). For the Nyquist plot, the real Z'-axis intercept was close to the ohmic resistance (R) of a membrane. The accelerated durability test was conducted by using CV method. The CV test was repeated in the potential range of 0.05 to 1.20 V vs. RHE and 100 mV s^{-1} for 5000 cycles with fully humidified H_2/N_2 gas supplied to anode and cathode, respectively. After ADT test, single cell performance and ECSA of samples were compared.
